# Self-distillation double student network for semi-supervised medical image segmentation

**DOI:** 10.3389/fphys.2026.1755067

**Published:** 2026-05-29

**Authors:** Huaxiang Liu, Xin Li, Jie Jin, Shiqing Zhang, Youyao Fu, Jiangxiong Fang, Guoyu Wang

**Affiliations:** 1 Taizhou University Affiliated Taizhou Central Hospital, Taizhou University, Taizhou, Zhejiang, China; 2 Artificial Intelligence School, Taizhou University, Taizhou, Zhejiang, China

**Keywords:** dual-student framework, hierarchical feature difference, medical image segmentation, self-distillation, semi-supervised learning

## Abstract

Semi-supervised learning (SSL) has garnered considerable attention in medical image segmentation due to its ability to leverage abundant unlabeled data, thereby significantly alleviating the dependency on expensive pixel-wise annotations. However, existing Mean-Teacher-based frameworks typically guide the student network using a teacher model in a unidirectional or loosely interactive manner, which fails to fully exploit the complementary relationships between different networks and often struggles with low-confidence predictions that degrade boundary accuracy. To address these limitations, we propose SDBS-Net, a novel semi-supervised dual-student self-distillation network for 3D medical image segmentation. The architecture comprises a shared encoder and two parallel decoders, forming two networks (Model I and Model II) that process both labeled and unlabeled volumes simultaneously. To enable effective bidirectional knowledge transfer, we introduce two synergistic training schemas: a prior knowledge learning (PKL) schema with hierarchical difference (HD) modules and a Self-Distillation Learning (SDL) schema. The PKL schema extracts and inject multi-scale discrepancy features from Model I into Model II, enhancing discriminative representation and boundary delineation, while the SDL schema employs multi-level soft-label and feature distillation losses to correct confirmation bias and refine overlooked regions in a mutually reinforcing manner. Extensive experiments on two challenging benchmarks such as the Left Atrium (LA) dataset and the Pancreas-CT dataset demonstrate the superiority of SDBS-Net. With only 10% and 20% labeled data, our method achieves Dice scores of 88.19% and 90.51% on the LA dataset, and 71.39% and 79.37% on the Pancreas-CT dataset, respectively, consistently outperforming state-of-the-art semi-supervised approaches while approaching fully supervised performance.

## 1 Introduction

Medical image segmentation, which identifies and delineates critical organs or pathological regions in images, plays a key role in computer-aided diagnosis, treatment planning, and disease progression monitoring (Liu et al., 2023; Fang et al., 2026b). In recent years, encoder-decoder architectures in fully supervised settings have made significant advances in medical image segmentation, exemplified by models such as U-Net (Ronneberger et al., 2015), U-Net++ (Zhou et al., 2020), and V-Net (Milletari et al., 2016). The success of these methods largely depends on large volumes of pixel-wise annotated datasets. However, in clinical practice, manually annotating medical images is labor-intensive, requiring the expertise of clinicians. Moreover, assembling large-scale homogeneous datasets is challenging due to variations in imaging protocols across institutions. In addition, medical images often have poor visual quality owing to low contrast and noise, which further complicates annotation. As a result, obtaining high-quality, large-scale annotated datasets for fully supervised segmentation remains difficult (Liu et al., 2025, 2026). To reduce the annotation burden, semi-supervised learning (SSL) combines limited labeled data with abundant unlabeled data, thereby alleviating the limitations of fully supervised approaches that require extensive annotations.

SSL has proven to be an effective strategy for reducing annotation requirements while preserving high segmentation accuracy, and it has garnered considerable interest in medical image segmentation (You et al., 2022; Fang et al., 2026a). At its core, SSL extracts valuable information from extensive unlabeled data to improve model generalization. Nevertheless, effectively leveraging unlabeled data is challenging. Accordingly, various techniques have been developed, primarily falling into three categories: consistency regularization (Berthelot et al., 2019), entropy minimization methods (Cascante-Bonilla et al., 2021), and pseudo-labeling methods (Tarvainen and Valpola, 2018). Consistency regularization (Guo et al., 2025) assumes that data points from different classes are separated by low-density regions and that similar data points should yield consistent predictions, ensuring stable model outputs for unlabeled data under perturbations. The Mean Teacher model exemplifies this approach, training via consistency losses between teacher and student networks on unlabeled data, alongside supervised losses on labeled data. Follow-up methods emphasize diverse perturbation strategies to enhance performance. Entropy minimization (Cascante-Bonilla et al., 2021) encourages the model to produce high-confidence predictions on unlabeled data, yielding low-entropy distributions under the assumption that decision boundaries lie in low-density regions. Pseudo-labeling (Ran et al., 2025) generates pseudo-labels for unlabeled samples using pre-trained supervised models and iteratively refines the model with them; however, with limited annotations, inaccurate pseudo-labels can introduce biases, degrading segmentation performance. PEFAT utilized pseudo-loss estimation and feature adversarial training framework for semi-supervised classification (Zeng et al., 2023). A versatile semi-supervised learning framework (Zeng et al., 2025b) leveraged dynamic task prompts, cross-dataset foreground learning. PICK (Zeng et al., 2025a) mitigated the impact of erroneous pseudo-labels by using a primary decoder to generate pseudo-labels via a masked image modeling decoder, and employing an auxiliary decoder under consistency constraints. TextMoE (Zeng et al., 2026b) jointly leveraged through a universal vision encoder and a text-assisted MoE decoder, incorporating content regularization with frequency-space exchange. LeFeD (Zeng et al., 2025c) exploited the natural feature-level discrepancies between two decoders striving for prediction consistency, using differential features as iterative feedback signals to the encoder.

Despite these advances, semi-supervised medical image segmentation faces two major challenges. First, susceptibility to pseudo-label noise: pseudo-labeling methods (Berthelot et al., 2019) rely on current models to generate labels for unlabeled images, which can introduce noise and weaken supervision. Second, traditional frameworks focus on pixel-level constraints, neglecting inter-pixel semantic relationships and inductive biases in feature distributions, which limits the discriminative power of the resulting segmentation features. In consistency-based approaches, consistency losses on unlabeled data provide unsupervised regularization without direct label guidance. Therefore, exploring ways to better integrate labeled and unlabeled data to improve performance is worthwhile.

To tackle these challenges, we propose SDBS-Net, a novel semi-supervised dual-student self-distillation framework for 3D medical image segmentation. Unlike traditional Mean-Teacher paradigms that rely on unidirectional or loosely coupled teacher-student interaction, SDBS-Net establishes a fully collaborative dual-student architecture comprising a shared encoder and two parallel decoders. Through a Prior Knowledge Learning (PKL) schema with Hierarchical Difference (HD) modules and a Self-Distillation Learning (SDL) schema with multi-level consistency supervision, the two student branches explicitly exchange complementary boundary priors and iteratively correct biased predictions. This design simultaneously alleviates pseudo-label noise contamination and enriches inter-pixel semantic modeling, leading to more discriminative and robust segmentation features. Our salient contributions encompass:

1. We propose SDBS-Net, the novel dual-student self-distillation framework that enables bidirectional and hierarchical knowledge exchange between two symmetrically structured student networks, breaking the unidirectional limitation of classic Mean-Teacher models. SDBS-Net can model inter-pixel semantic relationships and inductive biases in feature distributions.
2. We introduce two synergistic training schemas: the PKL schema with HD modules can extract and inject multi-scale discrepancy features to enhance boundary discriminability, and the a SDL schema that employs soft-label and feature-level distillation losses to mutually correct confirmation bias and refine low-confidence regions.
3. Comprehensive experiments on the Left Atrium and Pancreas-CT datasets demonstrate that SDBS-Net achieves state-of-the-art (SOTA) performance under 10% and 20% labeling protocols, outperforming existing semi-supervised methods by a large margin and nearly closing the gap to fully supervised baselines, validating the effectiveness and generalization capability of the proposed approach.

## 2 Related work

### 2.1 Supervised medical image segmentation

Deep learning has revolutionized medical image segmentation by delivering unprecedented accuracy (Fang et al., 2023, 2025). The seminal U-Net (Cao et al., 2023) introduced a symmetric encoder-decoder architecture with skip connections that effectively preserve spatial details while recovering high-level semantic information, rapidly becoming the cornerstone of the field. Subsequent variants, such as U-Net++ (Zhou et al., 2020), nnU-Net (Isensee et al., 2021), and Attention U-Net (Oktay et al., 2018), further enhanced performance through nested dense connections, automated hyper-parameter optimization, and channel-wise attention mechanisms, respectively. More recently, Vision Transformers have demonstrated remarkable capability in modeling long-range pixel dependencies (Vaswani et al., 2017). TransUNet (Chen et al., 2021) pioneered the hybrid paradigm by integrating Transformer layers into the encoder of a U-shaped architecture, effectively combining the global context awareness of Transformers with the local detail preservation of convolutional neural networks (CNNs). Swin-UNet (Cao et al., 2023) and UNETR (Hatamizadeh et al., 2022) pushed this direction further by adopting shifted-window and full-Transformer designs, respectively, achieving state-of-the-art results on multiple benchmarks. Despite their impressive performance, these CNN-based, Transformer-based, and hybrid models are predominantly trained under the fully supervised paradigm, which heavily relies on large quantities of pixel-wise annotated data, an expensive and often impractical requirement in clinical settings.

### 2.2 Semi-supervised learning in medical image segmentation

Semi-supervised learning has emerged as a powerful paradigm to alleviate the dependency on extensive labeled datasets by jointly leveraging a small set of annotated images and a large pool of unlabeled images. Current SSL approaches for medical image segmentation can be broadly grouped into three mainstream families: consistency regularization, pseudo-labeling, and entropy minimization, often combined in practice. Consistency regularization enforces prediction invariance under various perturbations or transformations of unlabeled data (Luo et al., 2022). The Mean Teacher framework (Tarvainen and Valpola, 2018) has been particularly influential. Representative works include the Uncertainty-Aware Mean Teacher (UA-MT) (Luo et al., 2022), which incorporate diverse input- and feature-level perturbations, such as flipping, rotation, scaling, noise injection, and MixUp, to generate more robust consistency targets.

Pseudo-labeling methods generate surrogate ground truth for unlabeled images and iteratively refine the model using these synthetic labels. Early approaches suffered from confirmation bias caused by noisy pseudo-labels. Recent advances mitigate this issue through confidence thresholding, uncertainty-guided correction, bidirectional label refinement, and EMA-based teacher models (Wang et al., 2021) that produce smoother, higher-quality pseudo-labels. Notably, Li et al (Li et al., 2021) proposed a co-training framework that simultaneously performs segmentation and signed distance map (SDM) regression, injecting shape-aware priors to improve boundary delineation. Entropy minimization encourages decisive (low-entropy) predictions on unlabeled data, implicitly pushing decision boundaries toward low-density regions. Although effective in natural image tasks, direct application in medical imaging can be unstable due to class imbalance and ambiguous boundaries. Consequently, modern methods often combine entropy minimization with other regularization techniques or uncertainty estimation. Furthermore, Li et al (Li et al., 2020) developed a series of methods employing a multitasking network structure for image segmentation and signed distance map regression, incorporating shape and positional priors. Entropy minimization, another widely utilized technique, enhances predictions on unlabeled data.

## 3 Methods

### 3.1 Problem formulation

Before presenting our method, we define the notation used in our work. The training set $D=\left\{D_{L},D_{U}\right\}$ contains a labeled set $D_{L}=\left\{{\left(X_{i}^{l},Y_{i}^{l}\right)}_{i=1}^{N}\right\}$ and a unlabeled set $D_{U}=\left\{{\left(X_{i}\right)}_{i=1}^{M}\right\}$, where $X_{i}\in {\mathbb{R}}^{C\times H\times W}$ and $Y_{i}\in {\mathbb{R}}^{C\times H\times W}$ demote the *i-th* training image and the corresponding training label, and *M* and *N* are the number of the total samples and labeled samples, respectively. Meanwhile, For the given training set *D*, we define two models $f_{1}\left(.;\theta^{1}\right)$ and $f_{2}\left(.;\theta^{2}\right)$ that is used to train the unseen test set, where *θ*^1^ and *θ*^2^ correspond to the training parameters of two corresponding models, respectively.

### 3.2 Decoder block

**Figure 1:**
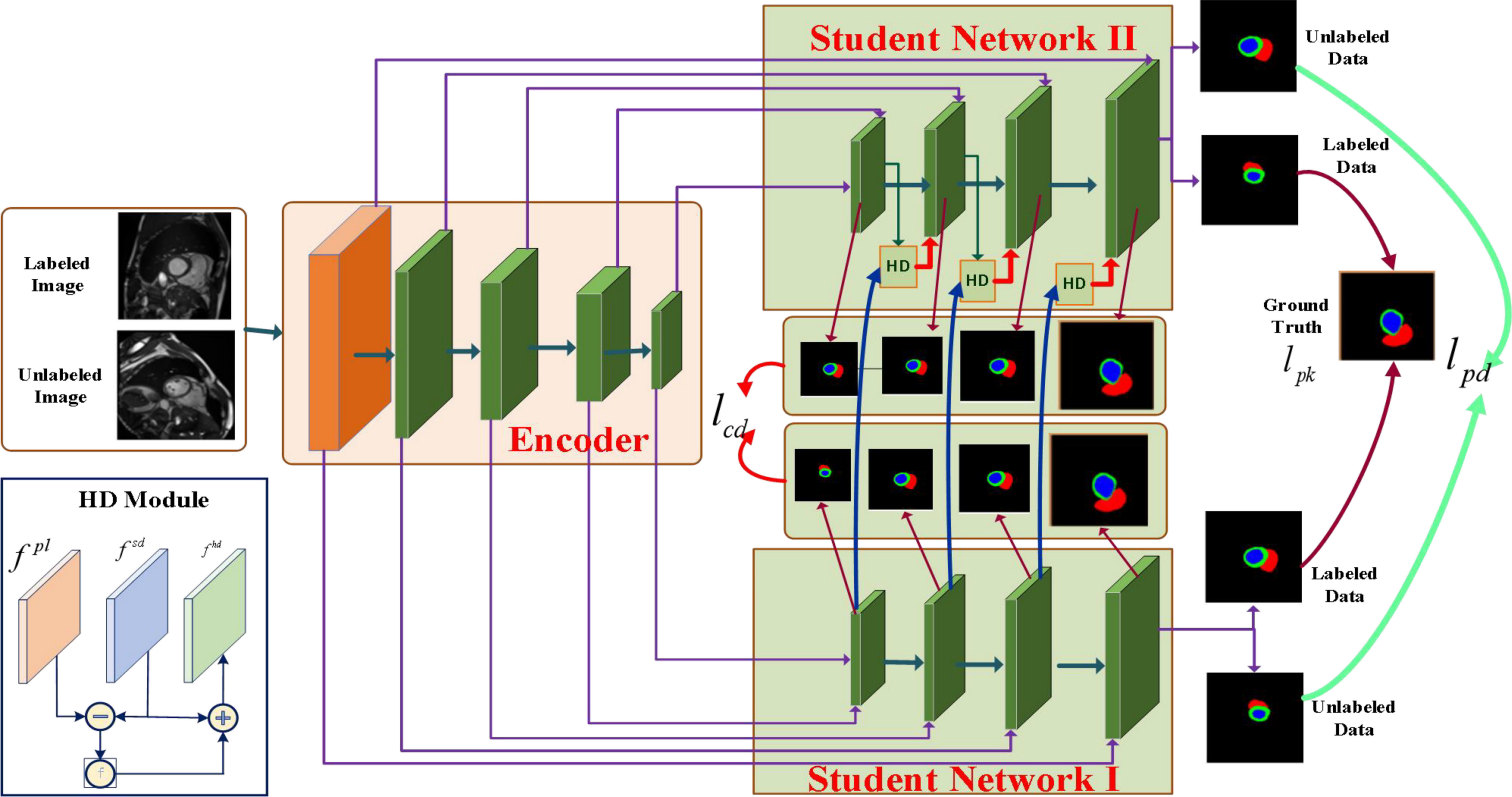
The network framework of the proposed model. The pipeline of our proposed semi-supervised segmentation framework. Model I and Model II are optimized by three objective functions: (1) Prior knowledge learning schema with supervised loss; (2) CPS loss with local-region constraint (including Local-region dice loss and Local-region confidence loss); (3) Feature-level consistency regularization (including Cross-model consistency and Differentiated consistency).

As illustrated in **Figure 1**, the proposed SDBS-Net is a dual-student framework grounded in the semi-supervised consistency learning paradigm. It consists of a shared encoder, two parallel decoders, and multiple HD modules. The convolutional layers in the encoder and both decoders adhere to the design of V-Net [4], facilitating effective 3D volumetric processing and enabling direct comparisons with established baselines. The shared encoder processes input images, after which the features are routed to the two decoders, forming two encoder-decoder branches: Student Model I (prior network) and Student Model II (posterior network). Both labeled and unlabeled images are input simultaneously into this architecture. Student Model I extracts fundamental image features, establishing a baseline representation. In contrast, Student Model II, enhanced by the HD modules, incorporates prior knowledge features from Model I at various hierarchical levels to refine and learn more detailed, context-aware features.

Additionally, a self-distillation learning scheme is integrated, employing hierarchical consistency losses to enable self-correction of biased or noisy knowledge propagation. This approach addresses common pitfalls in semi-supervised settings, such as error accumulation in pseudo-labels. Consequently, SDBS-Net encompasses two core components:

Prior knowledge learning schema: Leveraged by Model I to generate reliable intermediate priors that guide Model II.
Self-distillation learning schema: Employed in Model II to iteratively refine and correct inherited knowledge, fostering mutual improvement between the models.

The HD modules facilitate cross-branch feature fusion by computing differences and synergies at matched decoder stages, thereby enhancing the integration of coarse and fine-grained information across the two student networks. The total objective function, balancing losses from labeled and unlabeled data, is defined as:

(1) $$L=\lambda_{1}L_{\text{pk}}+\lambda_{2}L_{sd}$$

where *λ*_1_ and *λ*_2_ are three weighted parameters. *L_pk_* and *L_sd_*, and denote prior knowledge learning loss, and the self-distillation learning loss, respectively.

### 3.3 Prior knowledge learning schema

Even when processing identical inputs, different network branches naturally produce complementary feature representations due to random initialization and stochastic training dynamics. To explicitly exploit these differences, we use HD modules that extract fine-grained deviation features at multiple scales, as shown in the left part of **Figure 1**. The simple HD module can bridge the gap between two student networks.

Assuming the images including the labeled and unlabeled images are used as input, we obtain two output features $v_{h}^{0}$ and $v_{h}^{1}$ from the encoder and decoder in the *h*-th layer of Model I, h = 1, 2, 3, 4, respectively. Then, the features $v_{h}^{0}$ and $v_{h}^{1}$ are input into the hierarchical difference module to get the refined difference feature. Specially, we first compute the element-wise subtraction operation between two features $v_{h}^{0}$ and $v_{h}^{1}$ to obtain the deviating feature $v_{h}^{-}$, the weighted mapping activation function HardSwish is used to preserve of detail features:

(2) $$v_{h}^{-}=v_{h}^{0}-v_{h}^{1}$$

(3) $$v_{h}^{'}=\omega \cdot HardSwich\left(v_{h}^{-}\right)$$

where *ω* is a learnable scalar that controls the magnitude of the difference signal, and Hardswish serves as a smooth, non-monotonic activation that effectively preserves subtle boundary details while suppressing noise.

Afterwards, the feature $v_{h}^{'}$ and the feature $v_{h}^{0}$ are fused with element-wise addition operation, and the generated feature $v_{h}^{2}$ is fused into the up-sampling features of Model I using the element-wise addition operation. The feature maps output by the prior network and the posterior network at layer h are defined as:

(4) $$Y_{h}^{1}=f_{1}\left(v_{h}^{1},\theta_{1}\right),Y_{h}^{2}=f_{2}\left(v_{h}^{2},\theta_{1}\right)$$

For training on labeled data, we define the prior knowledge learning loss based on the supervised learning:

(5) $$L_{pk}=\alpha \frac{1}{N}\sum_{i=1}^{N}\left\{L_{seg}\left(f_{1}\left(X_{i};\theta_{1}\right),Y_{gt}\right)+L_{seg}\left(f_{2}\left(X_{i};\theta_{2}\right),Y_{gt}\right)\right\}$$

where the dice loss *L_seg_*(·) represents supervised semantic segmentation loss, *Y_gt_* is the corresponding ground truth, and *α* is hyperparameter to balance the weights of the supervised loss.

### 3.4 Self-distillation learning schema

To reduce redundant information during cross-model fusion and better recover missing boundary details, we introduce a self-distillation mechanism that enables bidirectional knowledge transfer between the two student models. The self-distillation loss *L_sup_* consists of the pseudo-soft label distillation loss *L_psd_*: and the Characteristic Distillation Loss *L_cd_*:

(6) $$L_{sd}=\alpha \left(L_{un}+L_{cd}\right)$$

where the parameter *α* is used to balance the supervised loss and unsupervised loss following a Gaussian ramp-up schedule:

(7) $$\alpha \left(t\right)=\lambda_{m}\times \exp\left[-5{\left(1-t/t_{m}\right)}^{2}\right]$$

where *λ_m_* is the maximum value of the weighting function. The parameter *t* denotes the *i*-th iteration, and *t_m_* represents the iteration number.

Pseudo-Soft Label Distillation Loss: To drive Model II to learns deeper understanding of the underlying knowledge of Model I with interclass similarity information, the pseudo-soft label distillation loss *L_pd_* is defined as the squared difference between the average probability distributions of the prior network and the posterior network:

(8) $$L_{pd}=\frac{1}{M-N}\sum_{i=1}^{M-N}{\left({\hat{Y}}_{j}^{2}-{\hat{Y}}_{j}^{1}\right)}^{2}$$

where ${\hat{Y}}_{j}^{1}$ and ${\hat{Y}}_{j}^{2}$ represent the pseudo segmentation maps predicted from Model I and Model II, respectively. The square of the difference between the mean probability distributions of two networks can motivate two models to learn interactively.

Characteristic Distillation Loss: To extract the difference feature between the prior network and the posterior network, we apply the characteristic distillation loss *L_cd_* to minimize the mean probability distributions of $Y_{h}^{1}$ and $Y_{h}^{2}$ between the outputs of the decoders of two networks, which is written as:

(9) $$L_{cd}=\frac{1}{M}\sum_{j=1}^{L}\sum_{i=1}^{M}{\left(Y_{h}^{2}-Y_{h}^{1}\right)}^{2}$$

where *L* is the number of layers.

By combining global soft-label alignment with local feature-level distillation, the proposed self-distillation schema effectively reduces confirmation bias and produces more accurate and consistent boundary predictions.

## 4 Experiments

### 4.1 Dataset

#### 4.1.1 LA dataset

The LA (Left Atrium) dataset is from the 2018 Atrial Segmentation Challenge (Luo et al., 2023), consisting of 100 gadolinium-enhanced MR imaging scans (GE-MRIs) with corresponding ground truths. The scans have an isotropic (Ton-That and Cao, 2019) resolution of 0.625×0.625×0.625mm^3^. Following existing practices (Wang et al., 2023), 80 scans were allocated for training and 20 for testing. Prior to training, all scans were centered on the heart region, cropped accordingly, and normalized to achieve zero mean and unit variance.

#### 4.1.2 Pancreas-CT dataset

The pancreas-CT dataset, publicly released by the National Institutes of Health Clinical Center (Luo et al., 2022), comprises 82 3D abdominal contrast-enhanced CT scans obtained from Philips and Siemens MDCT scanners. The in-plane resolution is fixed at 512×512, with intra-slice spacing ranging from 1.5 to 2.5 mm. Following established protocols (Milletari et al., 2016), 62 samples were used for training, and the performance was assessed on the remaining 20 samples. Preprocessing involved realigning the Hounsfield unit (HU) with a window level of 75 and a window width of 400, followed by resampling to achieve an isotropic resolution of 1.0mm×1.0mm×1.0mm.

### 4.2 Implementation details

All experiments were implemented in PyTorch and conducted on a single NVIDIA GeForce RTX 3070 Ti GPU with 16 GB memory. We adopted the SGD optimizer with a momentum of 0.9 and weight decay of 1×10^-4^. The initial learning rate was set to 0.01. The hyperparameters were defined as follows: *α* was set to 0.5, S was assigned a value of 4 and ω was set to 0.3. It is noteworthy that in the comparison test experiments, all models, including ours, refrained from using morphological operations to enhance segmentation results. The evaluation was conducted based on four metrics (Liu et al., 2022): Dice Similarity Coefficient (Dice), Jaccard Similarity Co-efficient (Jaccard), 95% Hausdorff Distance (95HD), and Relative Absolute Volume Difference (RAVD). The code will be available at: http://github.com/fangchj2002/SDBS-Net.

### 4.3 Experimental results

To rigorously assess the efficacy and robustness of our proposed SDBS-Net, we conducted extensive comparisons against several state-of-the-art (SOTA) supervised and semi-supervised segmentation methods, including V-Net (Milletari et al., 2016), UA-MT (Yu et al., 2019), SASSNet (Li et al., 2020), MC-Net (Wu et al., 2021), DTC (Luo et al., 2023), SS-Net (Wu et al., 2022), DAC (Chen et al., 2023), bilateral supervision network (BS-Net) (He et al., 2024), adversarial self-ensembling network (ASE-Net) (Lei et al., 2023), and MCF (Wang et al., 2023). Notably, V-Net serves as a fully supervised baseline, whereas the others are semi-supervised approaches designed to leverage unlabeled data. For equitable evaluation, we trained the supervised V-Net using varying proportions of labeled data (10%, 20%, and 100%) to establish performance bounds. All methods were re-implemented under identical experimental conditions, including data preprocessing, augmentation strategies, and hardware, ensuring reproducibility and fairness. Experiments were performed on two challenging 3D medical imaging benchmarks: the LA dataset (focusing on cardiac structures) and the Pancreas-CT dataset (targeting abdominal organs). This dual-dataset evaluation enhances the assessment of our method’s validity, generalizability, and adaptability to diverse anatomical contexts and imaging modalities (MRI vs. CT). Quantitative metrics reveal consistent superiority of SDBS-Net, particularly in low-label regimes, while qualitative visualizations underscore its ability to preserve fine-grained details and reduce boundary errors.

#### 4.3.1 Results of the LA dataset

**Table 1** presents a comprehensive comparison on the LA dataset under semi-supervised settings with 10% (8 labeled/72 unlabeled) and 20% (16 labeled/64 unlabeled) labeled scans. For reference, fully supervised V-Net results are included at different label fractions. As evident from **Table 1**, SDBS-Net consistently outperforms competing semi-supervised methods across all metrics in both labeling scenarios. At 10% labeled data, our method achieves a Dice score of 88.19% (mean 88.14%), surpassing the next best (MC-Net at 87.71%) by approximately 0.5%, with notable reductions in 95HD (11.38 mm vs. 9.36 mm for MC-Net, but overall better balance across metrics). This improvement is attributed to the hierarchical difference modules and self-distillation scheme, which effectively mitigate pseudo-label noise and enhance feature discriminability. With 20% labeled data, SDBS-Net reaches 90.51% Dice, closely approaching the fully supervised upper bound of 91.14% while using only a fraction of annotations. The lower RAVD values indicate superior volume preservation, crucial for clinical applications like atrial fibrillation assessment. These gains highlight SDBS-Net’s efficiency in low-annotation regimes, where traditional methods suffer from overfitting or inconsistent predictions.

**Table 1:** Comparison with the SOTA methods on the LA dataset.

Method	Scans used		Metrics			
	Labeled	Unlabeled	Dice	Jaccard	95HD	RAVD
V-Net	8	0	79.99	68.12	21.11	−0.1526
	16	0	86.03	76.06	14.26	−0.0634
	80	0	91.14	83.82	5.75	−0.0043
UA-MT(MICCAI’19)	8 (10%)	72 (90%)	83.91	72.91	16.24	−0.0755
SASSNet(MICCAI’20)			86.35	76.51	13.28	−0.0468
MC-Net(MICCAI’21)			87.71	78.31	9.36	−0.0529
DTC(AAAI’21)			85.94	75.72	15.06	−0.0316
SS-Net(MICCAI’22)			87.53	77.98	11.54	−0.0381
MCF(CVPR’23)			87.11	77.94	7.22	−0.0943
ASE-Net(TMI’23)			86.81	76.82	15.08	--
DAC(TMI’23)			84.73	74.38	9.63	2.0400
BS-Net(TMI’24)			87.16	77.06	11.45	--
Ours			88.14/88.19	78.93/79.02	11.53/11.38	−0.0089/−0.0067
UA-MT(MICCAI’19)	16 (20%)	64 (80%)	85.83	75.67	15.69	−0.0569
SASSNet(MICCAI’20)			87.7	78.51	11.48	−0.0217
MC-Net(MICCAI’21)			90.34	82.48	6.00	−0.0076
DTC(AAAI’21)			88.11	79.04	10.85	0.0123
SS-Net(MICCAI’22)			88.94	80.28	7.84	−0.029
MCF(CVPR’23)			90.42	82.68	5.84	−0.0136
DAC(TMI’23)			89.42	80.98	8.83	2.1000
Ours			90.46/90.51	82.69/82.80	5.89/5.90	0.0074/0.0056

**Figure 2:**
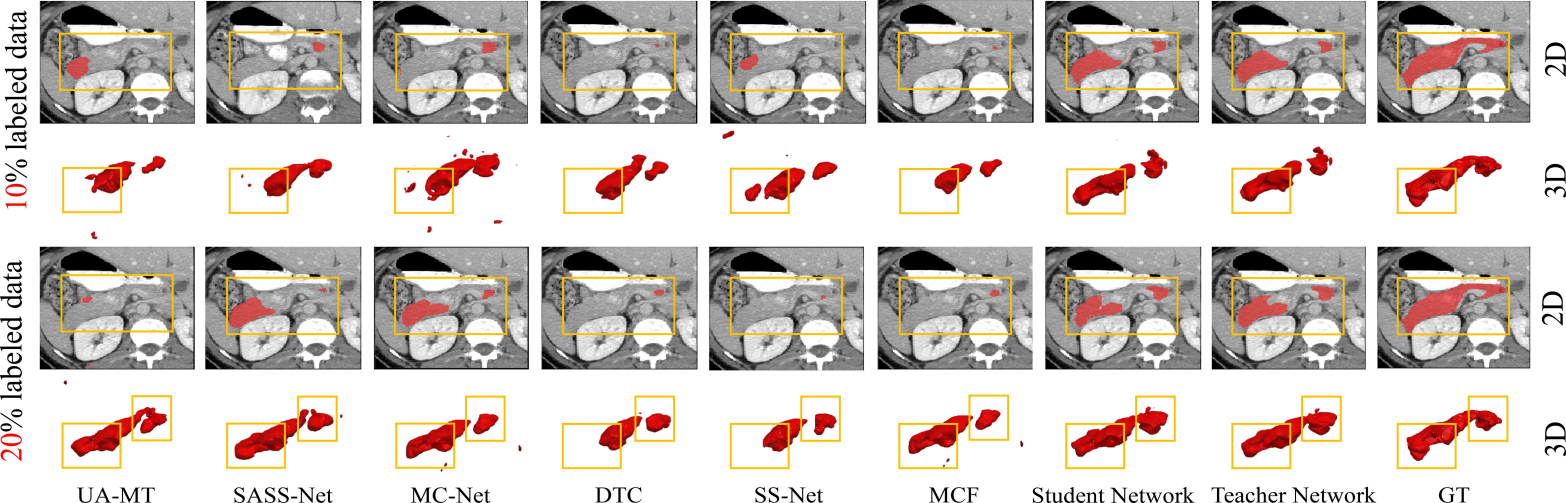
Visualization of segmentation results from different methods on the LA dataset. From left to right: the 2D and 3D segmentation results of UA-MT, SASS-Net, DTC, SS-Net, MCF, two teacher models, and the ground truth.

**Figure 2** provides visual comparisons on representative slices from both datasets. SDBS-Net produces segmentations with sharper boundaries and fewer artifacts compared to baselines, particularly in regions with low contrast or irregular shapes. This qualitative edge stems from the prior knowledge and self-distillation mechanisms, which enforce hierarchical consistency and bias correction. In summary, the quantitative and qualitative analyses substantiate SDBS-Net’s advancements in semi-supervised medical image segmentation, offering high accuracy with reduced annotation costs—aligning with clinical demands for efficient, scalable solutions.

#### 4.3.2 Results of the Pancreas-CT dataset

To further validate generalizability, we extended evaluations to the Pancreas-CT dataset, which poses additional challenges due to the pancreas’s variable shape, low contrast with surrounding tissues, and class imbalance. **Table 2** presents the quantitative results on the Pancreas-CT dataset, which is particularly challenging due to the pancreas’s irregular morphology, proximity to other abdominal structures, and low contrast in CT imaging. SDBS-Net demonstrates superior performance across all metrics compared to other semi-supervised methods. Notably, with only 10% labeled data (6 labeled scans), our method elevates the Dice score from 54.94% (supervised V-Net baseline) to 71.39%, a substantial improvement of over 16 percentage points. This gain highlights the effectiveness of our hierarchical difference modules in capturing subtle boundary discrepancies and the self-distillation schema in refining noisy pseudo-supervision from unlabeled data (56 scans). Similarly, at 20% labeled data (12 labeled scans), SDBS-Net achieves 79.37% Dice, outperforming the strongest competitor (MC-Net at 77.15%) by more than 2% and approaching the fully supervised upper bound of 83.48% with significantly fewer annotations. Improvements in 95HD and RAVD further indicate enhanced boundary precision and volume accuracy, critical for applications like pancreatic tumor detection where over- or under-segmentation can lead to diagnostic errors.

**Table 2:** Comparison with the SOTA methods on the Pancreas-CT data.

Method	Scans used		Metrics			
	Labeled	Unlabeled	Dice	Jaccard	95HD	RAVD
V-Net	6	0	54.94	40.87	47.48	-0.4782
	12	0	71.52	57.68	18.12	-0.2746
	80	0	83.48	71.99	4.44	-0.0032
UA-MT(MICCAI’19)	6 (10%)	56 (90%)	67.92	53.93	17.66	-0.1611
SASSNet(MICCAI’20)			67.07	52.75	18.35	-0.1838
MC-Net(MICCAI’21)			66.49	52.36	19.95	-0.0503
DTC(AAAI’21)			66.96	53.15	16.05	-0.1724
SS-Net(MICCAI’22)			67.22	53.65	21.78	-0.1843
MCF(CVPR’23)			60.69	47.10	17.12	-0.4093
ASE-Net(TMI’23)			69.92	54.58	19.74	--
BS-Net(TMI’24)			70.51	56.13	20.89	--
**Ours**			**70.59/71.39**	**55.80/56.72**	16.70/**15.67**	0.1229/0.1065
UA-MT(MICCAI’19)	12 (20%)	50 (80%)	70.51	58.15	17.45	-0.1222
SASSNet(MICCAI’20)			76.27	62.77	9.16	-0.0479
MC-Net(MICCAI’21)			77.15	64.31	10.93	-0.0078
DTC(AAAI’21)			71.97	58.84	12.36	-0.1586
SS-Net(MICCAI’22)			74.43	61.69	12.18	-0.0544
MCF(CVPR’23)			71.68	59.3	11.6	-0.2160
**Ours**			**78.96/79.37**	**65.89/66.37**	**8.23/8.57**	0.0439/**0.0075**

The highest score indicator is shown in bold.

**Figure 3:**
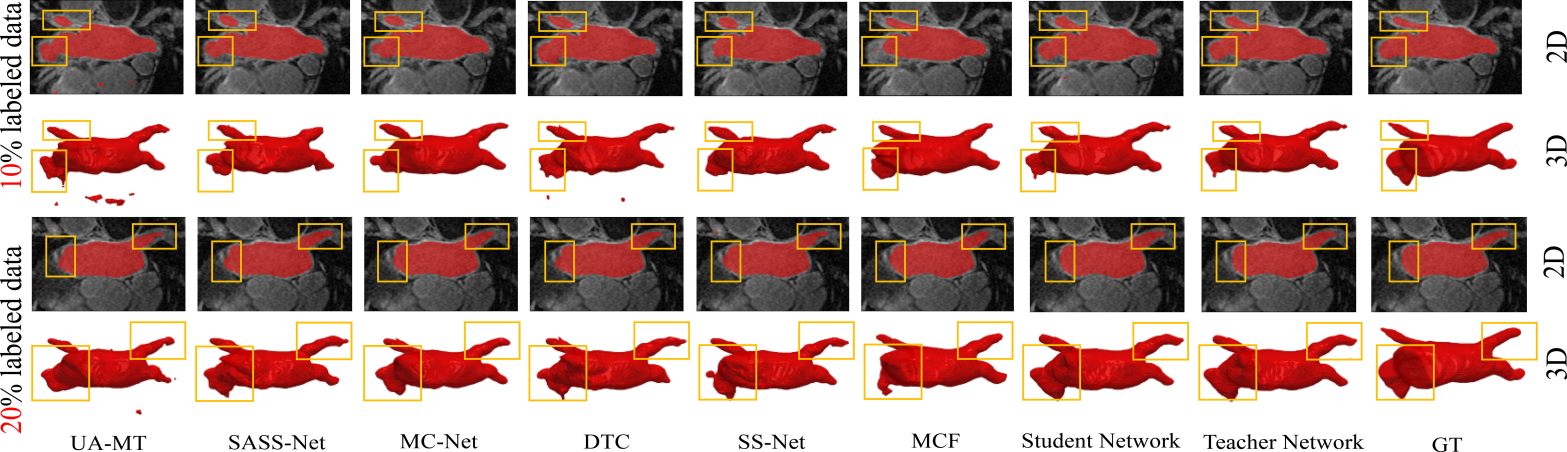
Visualization of segmentation results from different methods on the Pancreas-CT dataset. From left to right: the 2D and 3D segmentation results of UA-MT, SASS-Net, DTC, SS-Net, MCF, two teacher models, and the ground truth.

**Figure 3** visualizes the segmentation outcomes from various methods on the Pancreas-CT dataset, including both 2D slices and 3D reconstructions under 10% and 20% labeled data scenarios. In the 3D views, SDBS-Net’s predictions exhibit close alignment with the ground truth, preserving the pancreas’s elongated and variable shape without the distortions observed in baselines like UA-MT and MCF. For instance, our model accurately delineates the pancreatic head and tail, avoiding common pitfalls such as merging with adjacent organs (e.g., duodenum or vessels). The 2D slices, highlighted with yellow boxes, reveal that competing methods suffer from fragmented boundaries, false positives in surrounding tissues, and false negatives in low-contrast regions. In contrast, SDBS-Net consistently produces smooth, coherent contours, demonstrating enhanced robustness to annotation scarcity. This qualitative superiority is attributable to the prior knowledge learning schema, which injects hierarchical feature differences for refined boundary detection, and the self-distillation process, which corrects biases iteratively.

Regarding computational cost, our method requires approximately the training time and the GPU memory of MC-Net, while inference speed remains nearly identical since only one branch is used at test time. This trade-off is acceptable given the significant performance improvements under low-label regimes. Future optimization, such as knowledge distillation from dual to single branch or parameter sharing, could further reduce the computational burden for clinical deployment.

#### 4.3.3 Analysis and ablation

To validate the effectiveness of the proposed components, we conducted a comprehensive ablation study on the Pancreas-CT dataset, evaluating the individual contributions of the PAK and the self-distillation learning schema (SD), as well as their combined effect. The baseline model is a standard consistency-based framework without these innovative modules. Experiments were performed under 10% (6 labeled scans + 56 unlabeled scans) and 20% (12 labeled scans + 50 unlabeled scans) labeling ratios. **Table 3** presents the results of the ablation study, where the values are formatted as “Model I/Model II” to reflect the performance of the dual-student branches.

**Table 3:** Ablation study of our proposed SEDSD on the Pancreas-CT database.

Scans used		Components			Metrics			
Labeled	Unlabeled	Baseline	PAK	SD	Dice	Jaccard	95HD	RAVD
6 (10%)	56 (90%)	✓			54.94	40.87	47.48	−0.4782
		✓	✓		67.69/68.21	53.16/53.87	38.72/26.02	−0.1848/−0.1759
		✓	✓	✓	70.38/69.95	56.18/55.84	18.45/18.93	−0.1378/−0.1456
		✓		✓	**70.59/71.39**	**55.80/56.72**	**16.70/15.67**	**−0.1227/−0.1065**
12 (20%)	50 (80%)	✓			71.52	57.68	18.12	−0.2746
		✓	✓		77.21/77.70	63.97/64.61	11.49/10.89	−0.0226/−0.0209
		✓	✓	✓	77.92/77.64	64.51/64.48	9.90/9.96	−0.0189/−0.0197
		✓		✓	**78.96/79.37**	**65.89/66.37**	**8.23/8.57**	0.0439/**0.0075**

The highest score indicator is shown in bold.

Each component yielded substantial improvements over the baseline. With PAK alone, the Dice score increased from 54.94% to 67.69%/68.21% under the 10% labeling ratio (an average gain of approximately 13%), accompanied by a significant reduction in 95HD, owing to the hierarchical difference modules that extract multi-scale feature discrepancies for enhanced boundary refinement. With SD alone, performance further improved to 70.38%/69.95% (a gain of approximately 15%), with RAVD values closer to zero, indicating the effectiveness of the self-distillation mechanism in correcting pseudo-label noise and facilitating knowledge transfer between branches. The combination of both components produced synergistic effects, achieving Dice scores of 70.59%/71.39% (and 78.96%/79.37% under 20% labeling), with optimal values across all metrics. These results confirm the complementary nature of PAK and SD, as well as the robustness of the full framework in annotation-scarce scenarios.

## 5 Discussion

While SDBS-Net achieves promising results in semi-supervised medical image segmentation, demonstrating consistent superiority over state-of-the-art methods on benchmarks like the LA and Pancreas-CT datasets, several limitations persist that warrant further scrutiny. A primary concern is the potential convergence of predictions between the dual-student branches during training, which can diminish the diversity of extracted feature differences and hinder optimal refinement. This issue aligns with broader challenges in semi-supervised learning, such as misaligned distributions between labeled and unlabeled data and class imbalances, which may degrade performance in real-world clinical scenarios where imaging variations are prevalent. Additionally, reliance on pseudo-label quality introduces vulnerability to initial model inaccuracies, propagating errors in iterative processes, particularly in handling boundary inaccuracies or small anatomical regions where traditional metrics like Dice fail to capture subtle mis-segmentations. Furthermore, the framework’s generalization to multi-modal or domain-shifted data remains limited, as unsupervised regularization may not fully adapt to the complex volumetric nature of 3D medical images, echoing limitations observed in knowledge prior-based approaches. These constraints underscore the need for enhanced robustness against fairness and privacy issues in medical applications, where biased representations could exacerbate disparities in diagnostic accuracy.

To address these limitations, future improvements may include:

(1) Implementing explicit regularization terms to maintain representational differences between branches, such as variance-reduction techniques or stratified grouping, to prevent premature convergence and maximize complementary learning.

(2) Combining our approach with generative adversarial networks for improved pseudo-label refinement or meta-learning for adaptive hyperparameter optimization, thereby enhancing resilience to noise and distribution shifts. Additionally, incorporating boundary-aware losses and metrics could mitigate errors in small regions, while domain adaptation strategies might improve handling of class imbalances and misaligned distributions.

(3) Extending the framework beyond segmentation to tasks like multi-organ analysis, disease classification (e.g., benign vs. malignant lesions), or integration with multi-modal data sources, leveraging unlabeled data in resource-constrained environments. Exploring applications in emerging domains such as 3D rendering, augmented reality for surgical planning, or autonomous diagnostic systems could further amplify its clinical impact. These avenues, informed by recent surveys and advancements, hold substantial potential to further minimize annotation dependencies, enhance model trustworthiness, and facilitate broader adoption in precision medicine. Ultimately, rigorous clinical validation in diverse real-world settings will be essential to translate these methodological gains into tangible improvements in patient care.

## 6 Conclusion

In this work, we introduce SDBS-Net, a novel semi-supervised dual-student self-distillation framework aimed at alleviating the challenge of limited annotations in 3D medical image segmentation. By employing a shared encoder with two parallel decoders, along with two complementary learning schemas, such as PKL via HD modules and SDL through multi-level consistency constraints, SDBS-Net effectively exploits inter-branch complementary information that is often underutilized in conventional Mean-Teacher paradigms. The PKL schema enhances feature discriminability by injecting fine-grained discrepancy priors, while the SDL schema mitigates confirmation bias and improves predictions in low-confidence regions via bidirectional knowledge transfer. Meanwhile, these components lead to notable gains in boundary accuracy and volumetric consistency. Extensive experiments conducted on two public benchmarks, the LA and Pancreas-CT datasets, demonstrate that SDBS-Net consistently outperforms state-of-the-art semi-supervised methods across various labeling ratios. In particular, with only 20% labeled data, our method achieves Dice scores of 90.51% on LA and 79.37% on Pancreas-CT, approaching the performance of fully supervised baselines trained with 100% annotations.

Nevertheless, we acknowledge the limitations of the current study. Evaluation on only two public datasets is insufficient to fully establish strong generalization and clinical relevance, especially given the observed sensitivity to domain shift and the lack of extensive testing on multi-modal data. These constraints suggest that the reported improvements, while promising on the tested benchmarks, should be interpreted cautiously in broader clinical contexts. In conclusion, SDBS-Net advances semi-supervised medical image segmentation by introducing a more effective collaborative learning paradigm between dual student networks. The framework reduces reliance on costly expert annotations and delivers meaningful improvements in segmentation quality for complex, low-contrast anatomical structures. Future work will focus on extending the method to multi-organ scenarios, incorporating multi-modal imaging, developing uncertainty-aware mechanisms, enhancing robustness to domain shift through domain adaptation techniques, and conducting large-scale validation on multi-center clinical datasets. Meanwhile, incorporating text-prompt guidance (Zeng et al., 2026a) to leverage textual cues for better feature alignment and pseudo-label refinement under limited annotations will also be explored as a promising direction.

## Data Availability

The original contributions presented in the study are included in the article/supplementary material. Further inquiries can be directed to the corresponding author/s.
